# A Comprehensive Review of the Structure Elucidation of Tannins from *Terminalia* Linn.

**DOI:** 10.1155/2019/8623909

**Published:** 2019-11-15

**Authors:** Zihao Chang, Qiunan Zhang, Wenyi Liang, Kun Zhou, Ping Jian, Gaimei She, Lanzhen Zhang

**Affiliations:** School of Chinese Materia Medica, Beijing University of Chinese Medicine, Beijing 102488, China

## Abstract

**Objectives:**

Tannins with complex structures are important plant resources, which are abundant in the genus *Terminalia*. Various *Terminalia* species have been playing an important role in traditional medicine system. A systematic scoping review of *Terminalia* Linn. research literature for tannins was conducted to summarize the structures of tannins and analysis fragmentation pathway characteristics, which could provide references for the structural analysis of tannins from *Terminalia* Linn.

**Methods:**

After an update of the literature search up to September 2018, the terms of *Terminalia* in all publications were analyzed. Electronic searches were conducted in scifinder and PubMed, and the information from 197 articles in all with regard to the tannin structure study was extracted.

**Results:**

The compounds of 82 tannins from the genus *Terminalia* were reviewed. According to the structural differences, they can be divided into three categories, hydrolysable tannins, condensed tannins, and complex tannins, respectively. The fragmentation pathways of 46 identified tannins were analyzed, and the fragmentation rules of tannins were speculated according to different types.

**Conclusion:**

This review has attracted attention to the active substances in this species such as the tannins summarized in further study. How to improve the extraction and purification technology of tannins from genus *Terminalia* is an urgent problem to be solved.

## 1. Introduction

Plants of the genus *Terminalia* (family Combretaceae) are widely used in traditional medicine all over the world [1]. There are about 250 *Terminalia* species, of which at least 50 are used as food [2]. Many species have biological activities including antitumor, anti-inflammatory, wound healing, antifungal, antibacterial, and antiviral activities [3–7]. In particular, *Terminalia chebula*, an Indian species, is well noted as the king of plants in Ayurveda for its extensive medicinal use [8]. The plants mainly include tannins, polyphenols, triterpenoids, flavonoids, aliphatic compounds, and other active ingredients, among which tannins and polyphenols are the main constituents [9].

Tannins are a kind of polyphenolic compounds with complex structures in plants. They are classified into three groups on the basis of their structures: hydrolysable tannins, condensed tannins, and complex tannins. Usually, their molecular weights are greater than 500 Da. Tannins are widely distributed in various plants, and they are considered defensive molecules to protect plant tissues from herbivorous attacks because of their astringent taste [10]. It has been reported that several natural tannins and related compounds have various biological activities, including antioxidant, antitumor, hypolipidemic, hypoglycemic, and antibacterial activities [11–14]. Takashi Tananka isolated terflavin A and B, tercatain, and tergallagin from the leaves of *Terminalia catappa* Linn. in 1986 [15]. Since then, more than 82 tannins have been isolated from the fruits, barks, leaves, and galls in the plants of the genus *Terminalia*. The mass spectrometric data of these tannins and the structure analysis of the compounds are discussed. This review aims to provide references for the structure identification of tannin constituents in the plants of *Terminalia* Linn. In the further study of phytochemistry, the research field of medicinal activity of this important genus should be highlighted and guided.

## 2. Methods

### 2.1. Data Sources and Searches

Electronic searches were conducted in scifinder and PubMed for articles up to September 2018, using terms related to tannins, *Terminalia*, and MS. Searches were conducted with no date or language restrictions.

### 2.2. Eligibility and Selection

The titles and abstracts of 197 articles were screened, respectively, and the full text of the article was reviewed to obtain sufficient information. Any disagreements regarding the inclusion of articles were resolved through discussion and consensus.

### 2.3. Data Extraction

The final data extraction included the following five categories: (1) general characteristics (compound name, source, structure, and journal name); (2) MS data (compound name, ion Source, ion mode, fragments, and journal name); and (3) MS fragmentation pattern (fragmentation rules and journal name).

## 3. Results and Discussions

### 3.1. Tannins

Tannins are widely distributed in plants. They can be classified into three types according to their structural differences. Hydrolysable tannins are a group of compounds formed by phenolic acids and their derivatives through glycoside bonds or ester bonds with glucose or polyols. They are further divided into gallotannins containing only galloyl groups, ellagitannins containing hexahydroxydiphenoyl group(s), and hydrolysable tannin oligomers divided into dimers, trimers, and tetramers according to the number of glucose nuclei [16]. Condensed tannins are a class of compounds formed by the carbon-carbon bond polymerization of flavane-3-ol such as catechins or their derivative gallocatechin. Complex tannins are a class of compounds composed of flavane-3-ol, the unit of condensed tannins, and hydrolyzed tannins, which are partially linked by carbon-carbon bonds.

**Figure 1:**
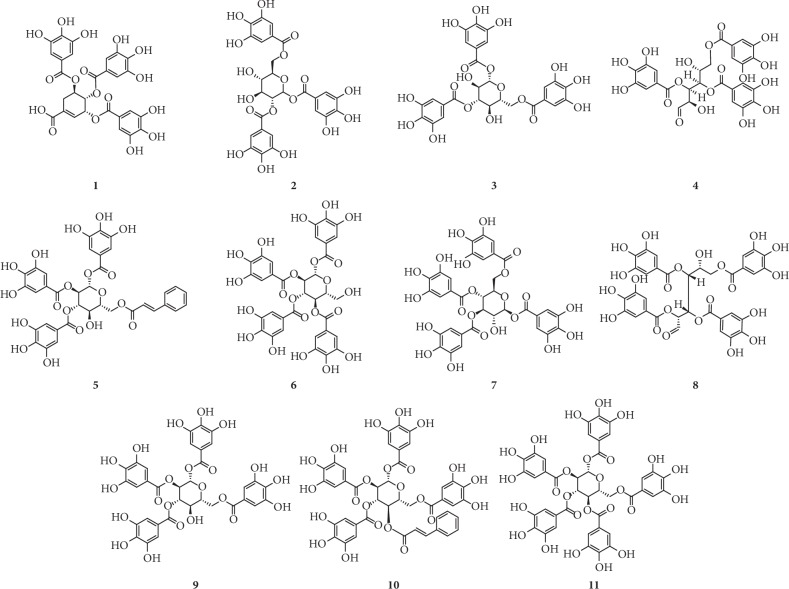
Structures of compounds **1**–**11**.

**Figure 2:**
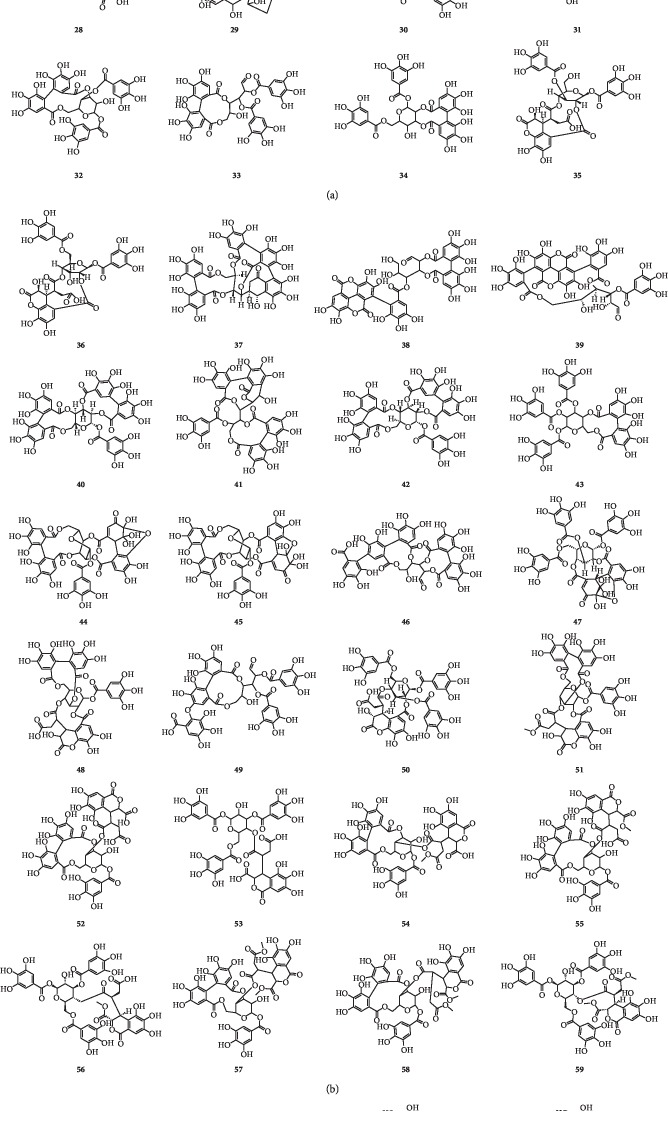
Structures of compounds **12**–**71**.

**Figure 3:**
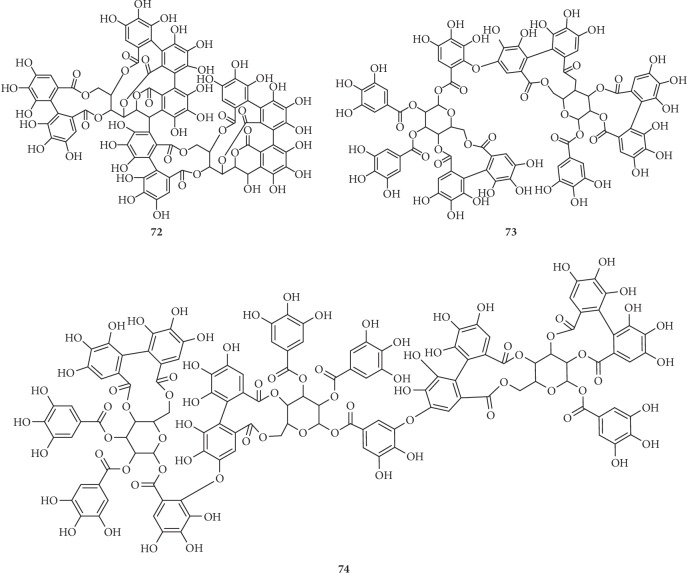
Structures of compounds **72**–**74**.

**Figure 4:**
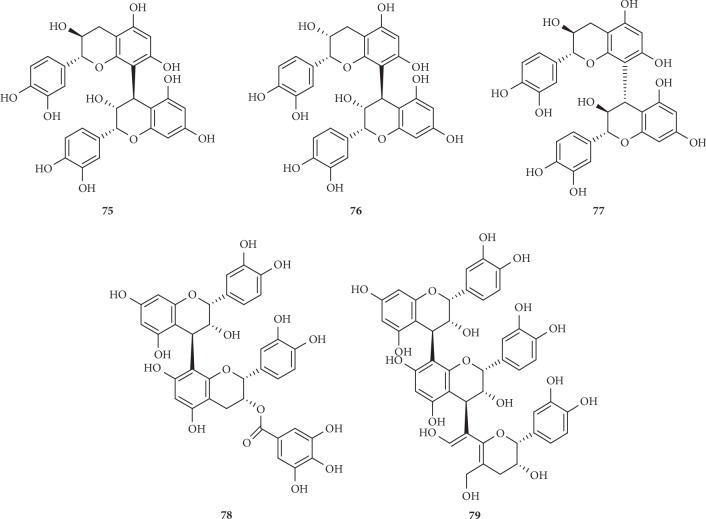
Structures of compounds **75**–**79**.

**Figure 5:**
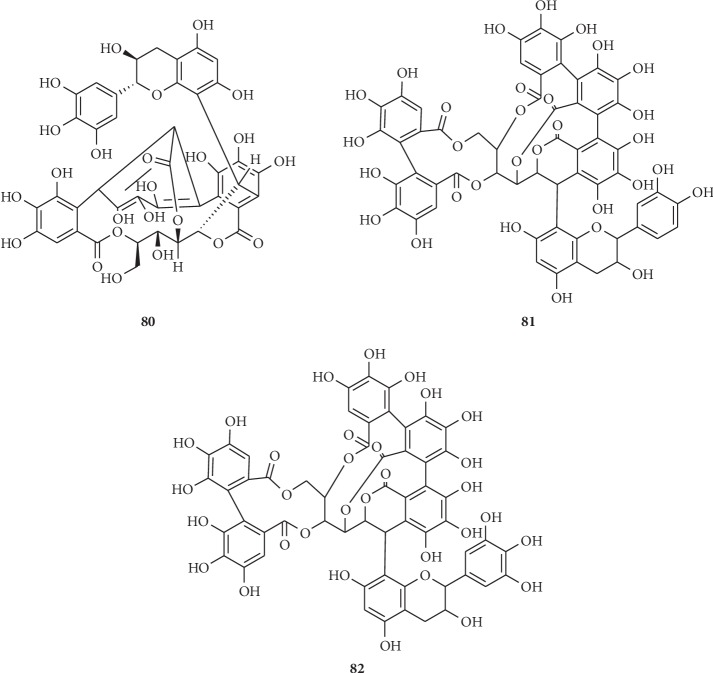
Structures of compounds **80**–**82**.

On the basis of the structural differences, they are divided into different types. Compounds **1**–**74** are hydrolysable tannins. Among them, compounds **1**–**11** (Figure 1) having only galloyl groups are gallotannins and compounds **12**–**71** (Figure 2) having hexahydroxydiphenoyl group(s) are ellagitannins. In addition, compounds **72** and **73** (Figure 3) possess two glucose nuclei, and compound **74** (Figure 3) possesses three glucose nuclei. Therefore, they are thought to be hydrolysable tannin dimers and hydrolysable tannin trimers, respectively. Meanwhile, compounds **75**–**79** (Figure 4) are condensed tannins. Compound **79** is further classified into condensed tannin trimers, and the others are condensed tannin dimers. Compounds **80**–**82** (Figure 5) possess the unit of condensed tannins and the unit of hydrolyzed tannins which are thought to be complex tannins. The names, corresponding plant resources, and related references of the compounds have been listed in Tables 1–5.

### 3.2. MS Data of Tannins

The MS data of the tannins from the genus *Terminalia* (family Combretaceae) are shown in Table 6 as summarized. According to the compiled MS data, this review provides a useful and fast way for the identification of tannins.

### 3.3. Fragmentation Pattern

#### 3.3.1. Gallotannins

Most gallotannins produce major fragment ions [M-H-170]^−^ and [M-H-152]^−^, which indicate the loss of gallic acid and galloyl residue. In addition, other fragment ions such as [M-H-170]^−^, [M-H-170-152]^−^, and [M-H-170-152-152]^−^ are produced owing to the sequential losses of galloyl group and gallic acid.

**Figure 6:**
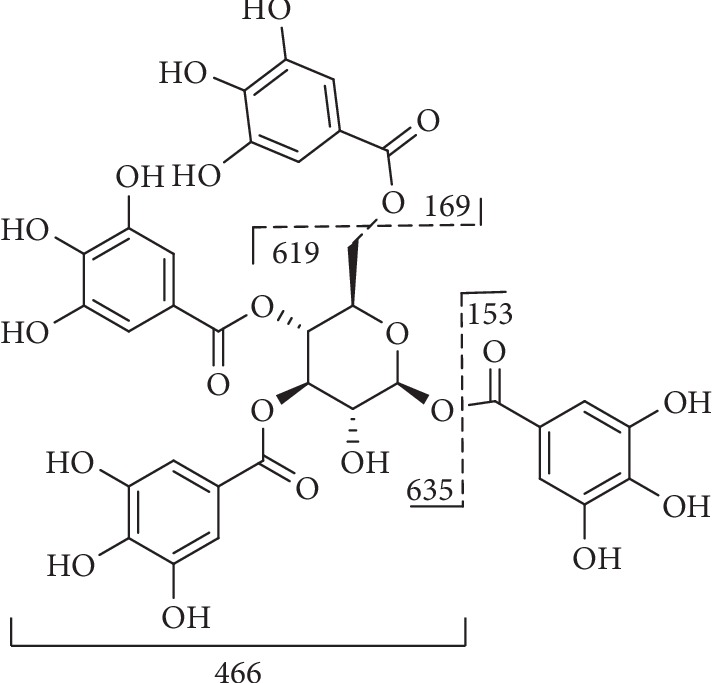
Fragmentation of compound **7**.

Compound **7** (Figure 6) gave the [M-H]^−^ ions at m/z 787 and displayed a fragmentation pattern similar to the successive neutral losses of gallic acids (170 Da) and galloyl radicals (152 Da). Due to the limited mass spectrometry information, it was difficult to distinguish the link position between galloyl groups and glucosyl unit [96].

**Figure 7:**
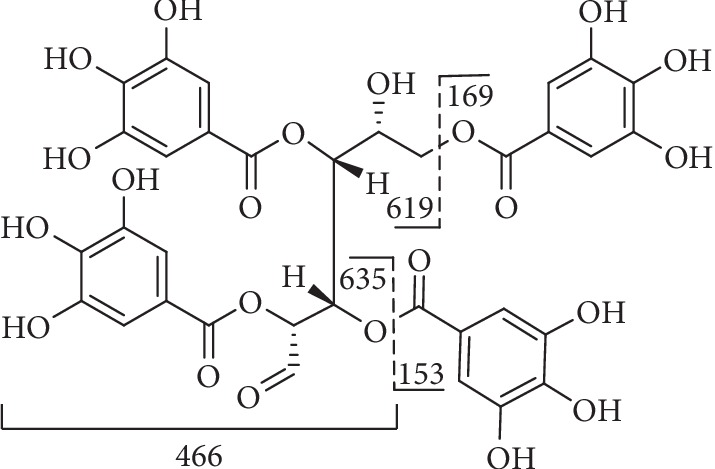
Fragmentation of compound **8**.

Compound **8** (Figure 7) is characterized by fragment ions at m/z 635, corresponding to the loss of a galloyl residue ([M-H-152]^−^) and at m/z 617 owing to the loss of a gallic acid group ([M-H-170]^−^) [97].

**Figure 8:**
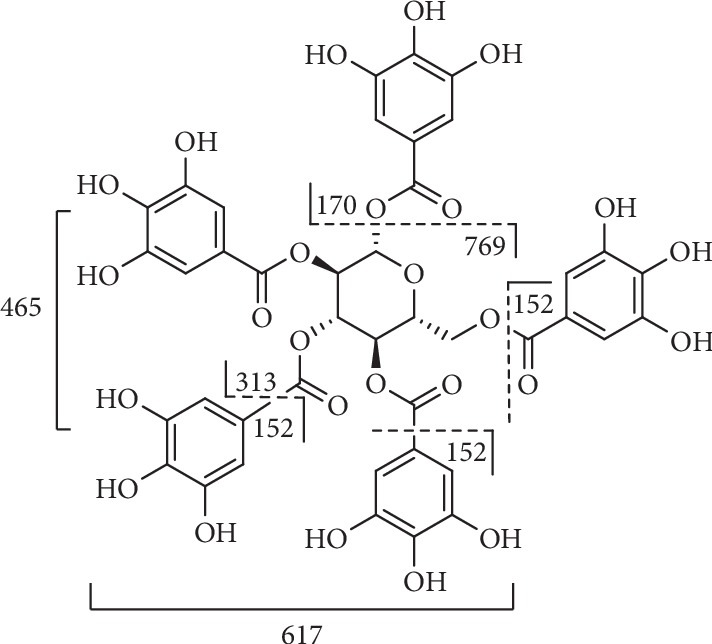
Fragmentation of compound **11**.

Compound **11** (Figure 8) with the [M-H]^−^ ion at m/z 939 and m/z 469 [M-2H]^2−^, showed typical fragments at m/z 769 [M-H-170]^−^, m/z 617 [M-H-170-152]^−^, m/z 465 [M-H-170-152-152]^−^, and m/z 313 [M-H-170-152-152-152]^−^ which corresponded to the sequential losses of galloyl group and gallic acid [108].

#### 3.3.2. Ellagitannins

Most ellagitannins produce major fragment ions [M-H-170]^−^, [M-H-170-162]^−^, and [M-H-302]^−^, which indicate the loss of gallic acid, galloylglucose group, and HHDP group. In addition, other fragment ions such as 151, 169, and 301 confirm the existence of galloyl group, gallic acid, and HHDP group, respectively.

**Figure 9:**
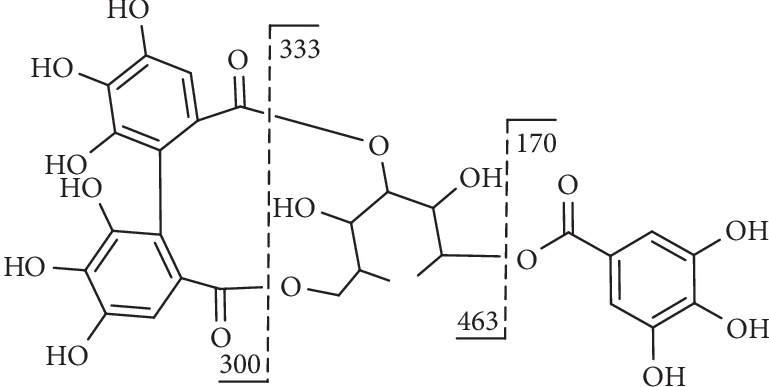
Fragmentation of compound **18**.

Compound **18** (Figure 9) presented [M-H]^−^ at m/z 633.0762 and MS^2^ fragments at m/z 463.0793 [M-H-152-H_2_O]^−^, which is consistent with sequential losses of galloyl and H_2_O and at m/z 300.9986 [M-H-152-180]^−^ owing to the loss of a galloyl unit with a hexose [117].

**Figure 10:**
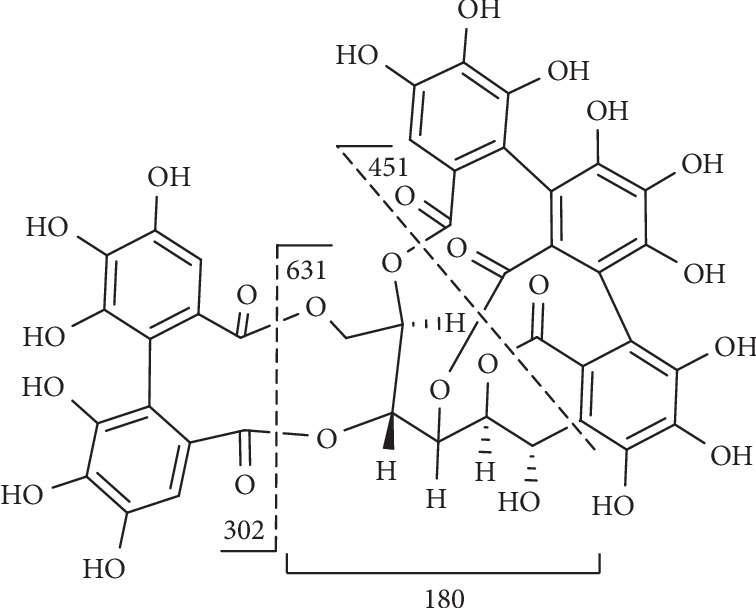
Fragmentation of compound **37**.

Compound **37** (Figure 10) displayed [M-H]^−^ at m/z 933 and MS^2^ ion at m/z 631 resulting from the loss of HHDP and presented MS^3^ ions at m/z 451 owing to the loss of glucosyl moiety and at m/z 301 which corresponded to the loss of galloyl-glucosyl moiety from the parent MS^2^ ion at m/z 631 [144].

**Figure 11:**
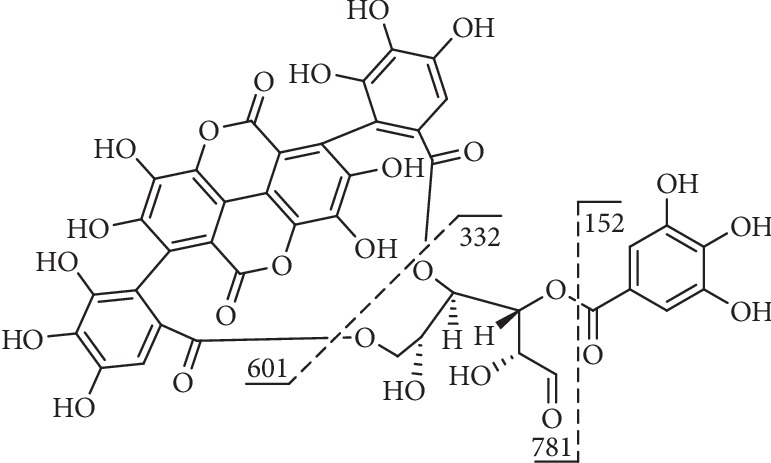
Fragmentation of compound **39**.

Compound **39** (Figure 11) had an [M-H]^−^ ion at m/z 933 and three mass fragments: one at 601 ([M-H-332]^−^) which corresponded to the loss of a galloylglucose unit and two others at m/z 781 ([M-H-152]^−^) which corresponded to the presence of a galloyl group and at m/z 721 after the cross-ring fragmentation of glucose ([M-H-152-60]^−^) [124].

**Figure 12:**
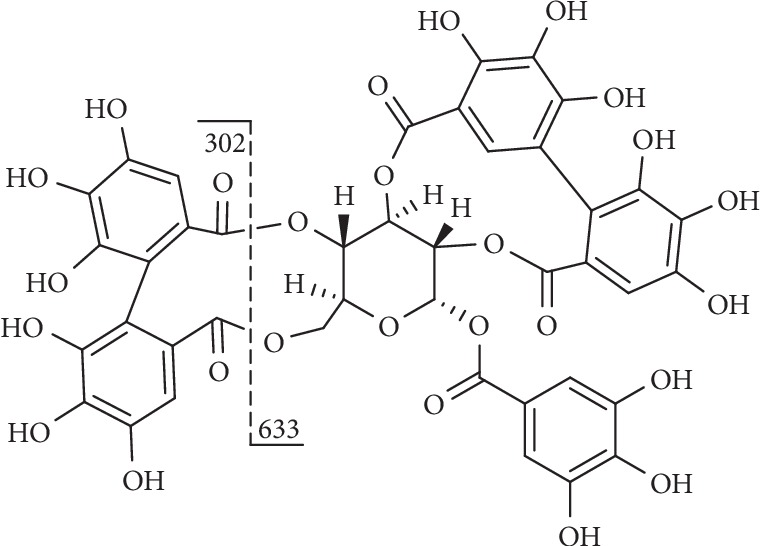
Fragmentation of compound **42**.

Compound **42** (Figure 12) displayed molecular anions at m/z 935 and produced fragments at m/z 633 ([M-H-302]^−^), corresponding to the loss of an HHDP group and at m/z 301 ([M-H-634]^−^), indicating the presence of HHDP (302 Da), gallic acid (170 Da), and glucosyl (162 Da) groups [129].

**Figure 13:**
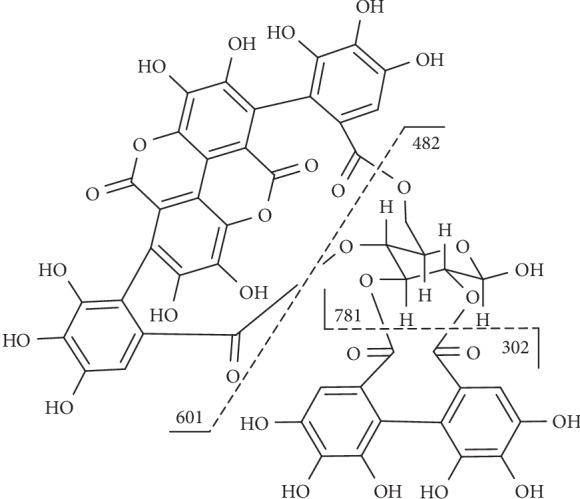
Fragmentation of compounds **62** and **63**.

Compounds **62** and **63** (Figure 13) are isomers, which had the same fragmentation behaviors, presented a same [M-H]^−^ ion at m/z 1083, and further produced ions at m/z 781 ([M-H-302]^−^), m/z 601 ([M-H-302-180]^−^), and m/z 301, demonstrating the existence of HHDP and gallagic acid groups [165].

#### 3.3.3. Condensed Tannins

Structurally significant product ions were produced by cleavages between monomeric subunits, which contain quinone methide (QM), heterocyclic ring fission (HRF), and retro-Diels–Alder (RDA) fragment ions.

QM fragmentation cleaves the single bond between subunits in B-type procyanidins to form a single quinone resulting in two possible product ions.

A second important structural fragmentation pathway for deprotonated procyanidins is heterocyclic ring fission (HRF), which results in the elimination of 1,3,5-trihydroxybenzene ([M-H-126]^−^).

Retro-Diels–Alder (RDA) fragmentation was distinguished by elimination of hydroxyvinyl benzenediol ([M-H-152]^−^), an extra water molecule ([M-H-152-18]^−^) simultaneously.

The dimeric procyanidins occur as the B-type procyanidins in nature, which contain four major isomers such as B1, B2, B3, and B4. We have sorted out compounds **75**–**77** which presented in *Terminalia* Linn. The three compounds presented the specific fragments of m/z 425 and 407, which corresponded to the characteristic fragmentations of procyanidin B-type dimmers [166].

**Figure 14:**
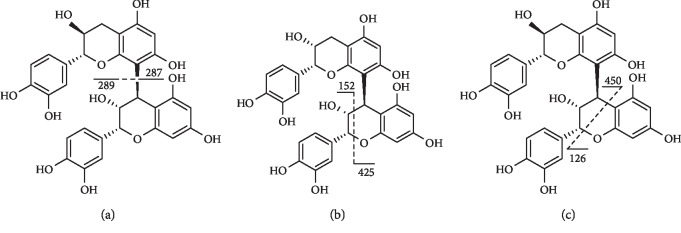
Fragmentation of compound **76**. (a) QM, (b) RDA, and (c) HRF.

Compound **76** (Figure 14) presented an [M-H]^−^ ion at m/z 577, with fragment ions at 425 ([M-H-152]^−^), originated from Retro Diels–Alder (RDA) fragmentation of the heterocyclic ring. The fragment at m/z 407 ([M-H-170]^−^) resulted from both RDA rearrangement and loss of water molecule [155].

Compound **77** had an [M-H]^−^ ion at m/z 577 which presented a Retro-Diels–Alder (RDA) product with a neutral loss of 152 ([M-H-152]^−^) and subsequently loss of a water molecule [M-H-152-18]^−^ [158].

**Figure 15:**
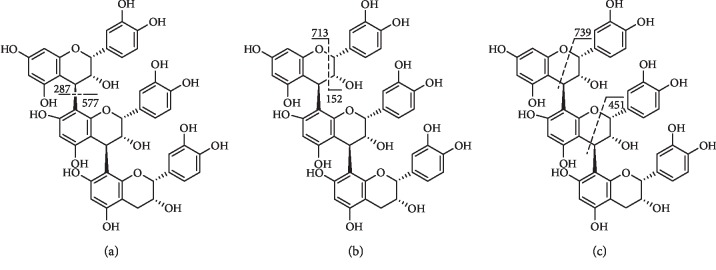
Fragmentation of compound **79**. (a) QM, (b) RDA, and (c) HRF.

Compound **79** (Figure 15) gave the [M-H]^−^ ion at m/z 865 and showed fragment ions at m/z 287/577 and m/z 575/289 due to QM fragmentation. The fragment at m/z 713/695 corresponded to RDA fragmentation and at m/z 425/407 owing to RDA fragmentation of the QM product ion of m/z 577. It also formed ions of m/z 739, m/z 451, and m/z 413 through HRF fragmentation [155].

#### 3.3.4. Complex Tannins

**Figure 16:**
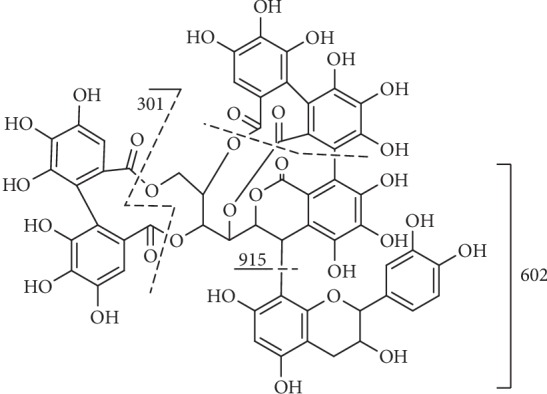
Fragmentation of compound **81**.

Compound **81** (Figure 16) had an [M-H]^−^ ion at m/z 1205 and other fragments at m/z 915, due to the loss of the substituent at C-1 of the vescalagin-derived nuclei structure and at m/z 613 resulting from the loss of the 4,6-hexahydroxybiphenoyl unit from the latter fragment and at m/z 301 which corresponded to the existence of ellagic acid [143].

## 4. Biological Activity

Natural compounds are important sources of drugs. More and more attention has been paid to the scientific investigation of natural bioactive compounds which may yield new compounds or leading compounds that can overcome the limitations of currently used drugs. At present, some achievements have been made in the study of tannins, but there are still some deficiencies. Tannins extracted from plants are often a collection of monomers of different kinds of tannins mentioned above. Their bioactivities are closely related to the action of these tannin monomers which need further studies. The reported biological activity of these tannins from the genus *Terminalia* (Family Combretaceae) was summarized briefly.

### 4.1. Antioxidant Activity

Ellagitannins such as compounds **18**, **48**, and **70** were found to be the major components in *Terminalia bellirica*, which exhibited the antioxidant and hepatoprotective activities [167]. Compounds **11**, **20**, **30**, **33**, and **43** exhibited great antioxidant activity in both chemical-based and cellular-based antioxidant assays, and compound **11** showed the highest cellular antioxidant activity [168]. Compound **11** has the highest potency for DPPH-, NO-, and ONOO-scavenging activity with IC50 ranging from 5 to 20 *μ*M, 0.20, and 0.06 *μ*M, respectively [169]. Compounds **33** and **43** showed the highest increase in GSH, and compound **30** produced the highest increase in SOD among four tannins [170]. Compounds **28** and **62** had *in vitro* antioxidant activity and *in vivo* antioxidative stress effects [171]. A lot of research showed that antioxidant compounds are related to a variety of oxidative stress-related diseases, such as cardiovascular diseases, neurodegenerative diseases, and cancer [172].

### 4.2. Anticancer Activity

It was confirmed that compound **18** could induce autophagy, apoptosis and ROS accumulation in gastric cancer cells *in vitro* [173]. IC50 values of HepG2, Molt-3, HL-60, NPC-BM1, HT 1080 and K562 were 1.42, 0.35, 0.12, 0.81, 1.02, 1.53 mg/mL *in vivo*, respectively [174]. A molecular mechanism study showed that the inhibition of the proliferation of ovarian cancer cells by compound **18** is mediated by blocking the TGF-beta/AKT/ERK/Smad signaling pathway [175]. Compound **11** could induce autophagy of HepG2, MCF-7, and A549 by activating MAPK 8/9/10 and JNK signaling pathways [176]. Compound **11** could also enhance GNMT promoter activity by downregulating MYC expression in hepatocellular carcinoma [177]. Compounds **70**, **62**, **63**, **42**, and **19** were isolated from *Terminalia calamansanoi* with the IC50 values of 65.2, 74.8, 42.2, 38.0, and >100 *μ*M, respectively, for HL-60 cells [178]. It was confirmed that protective effects of compound **20** against DNA damage are induced by different mutagens [179]. The chemopreventive effect of compound **62**/**63** on H-ras-induced transformation may be due to inhibition of intracellular redox status and activation of JNK-1/p38 [180]. Compounds **30**, **33**, **49**, and **68** could inhibit MCF-7/wt cell viability, and the inhibition ability is stronger with the number of functional units: hexahydroxydiphenoyl (HHDP) group [181]. Compound **50** was proven to have antiproliferative, proapoptotic, and antimigratory effects which are related to the PI3K/AKT and MAPK/ERK pathways [182].

### 4.3. Antimicrobial and Antivirus Activity

Compound **18** could inhibit biofilm formation, quorum sensing, and toxin secretion. This indicated that corilagin might be an effective antibacterial compound [183]. Compound **11** efficiently blocked entry of HCV of all major genotypes and also of the related flavivirus Zika virus [184]. Compound **11** could effectively inhibit the replication of RABV by the miR-455-5p/SOCS3/STAT3/IL-6-dependent pathway [185]. Compounds **28**, **44**, and **62** reduced the HCV replication [186] via a dual mechanism through preventing the formation of cccDNA and promoting cccDNA decay [187].

### 4.4. Antidiabetic Activity

It was confirmed that compound **18** can regulate diabetes, by exhibiting antidiabetic, antihyperlipidemic, and antioxidant properties in STZ-induced diabetic rats [188]. Compound **11** could maintain normal glycemia through the inhibitory action on alpha-amylases [189]. Compounds **22**, **48**, and **50** with the IC50 values of 690 *μ*M, 97 M, and 361 *μ*M could inhibit activity of mammalian intestinal maltase [53].

### 4.5. Other Therapeutic Activities

Compounds **20**, **30**, and **33** which have HHDP moiety decreased the ratio of MMPs/TIMPs to develop skin ageing [190]. Compound **48** was confirmed to inhibit TGF-beta 1-induced antifibrotic activity in choroid-retinal endothelial cells (RF/6A) [191] and inhibit TNF alpha induced proangiogenic and proinflammatory activities in retinal capillary endothelial cells [192].

The study of nanoparticles plays an important role in tannin activity and application. Bioavailability and bioactivity of a component are often altered once it is embedded into nanoparticles. Zheng Li fabricated the PPE with 16.6% (w/w) of punicalagin A, 32.5% (w/w) of punicalagin B, and a small amount of ellagic acid-hexoside and ellagic acid (1%, w/w). PPE-gelatin nanoparticle suspension had similar effects in inducing late stage of apoptosis and necrosis compared to PPE [193]. Guo-Bin Song fabricated a natural promising protein protective film through soluble dietary fiber (SDF)-tannin nanocluster self-assembly which characterized to possess a broad spectrum of antimicrobial properties and are beneficial to food preservation [194]. The field of nanoparticles plays an important role in the utilization of tannin activity with great development potential.

There is a lack of research on the interaction between proteins and tannins from *Terminalia* Linn., but the tannin extracted from persimmon fruits has been reported to have a high affinity to pancreatic lipase and possessed pancreatic lipase inhibition with IC50 of 0.44 mg/mL. Molecular docking showed that this interaction is mainly caused by the hydrogen bonding and *π*-*π* stacking [195]. It has been demonstrated that the very simple tannin methyl gallate was able to stack with itself or with caffeine [10]. The self-association of tannins should also take into account the interaction between tannins and proteins, as it governs their bioavailability. The interactions between tannin-tannin and tannin-protein are still unclear. Changes in protein bioactivity and structure induced by tannin binding need further studies.

Current limited metabolic studies showed that tannins are mainly metabolized as urolithins in the gut [196]. Urolithins are characterized to possess antitumor, antioxidative, and anti-inflammatory activities *in vitro*, which can be isolated and purified by high-speed counter-current chromatography. Urolithin A, a major punicalagin metabolite, could result in autophagy in SW620 colorectal cancer (CRC) cells at submicromolar concentrations [197]. It is very helpful for drug design to clarify the biotransformation of tannins *in vivo*.

Therefore, it is necessary to accelerate the development of the technical means for the analysis of bioactive compounds of natural medicines, so as to realize the large-scale development and utilization of tannin monomer compounds. The physiological activity of tannins has been fully confirmed, but the physiological mechanism of its various pharmacological effects is still not clear, limiting the development and utilization of tannins.

## 5. Conclusion

*Terminalia* species have been widely used in various traditional medical systems such as Siddha, Traditional Chinese Medicine (TCM), and Western, Southern, and Central African medicinal systems [8]. Apart from reports on the ethnopharmacological uses of many *Terminalia* species, few studies have carried out rigorous studies on the medical properties, mechanisms, and phytochemistry of this important genus. This may be due to the fact that tannins are the main active constituents in many *Terminalia* species. Tannins have strong polarity, high molecular weight, complex structure, active chemical properties, and are extremely difficult to crystallize which make them difficult to extract, separate, purify, and identify, and the quality standard is not easy to control. Therefore, they are so complex that they are not suitable for drug design and often overlooked as potential for drug discovery. Thus, how to improve the extraction and purification technology of tannins from genus *Terminalia* is an urgent problem to be solved. Researchers need to further determine the structure-activity relationship between tannins and their functions, clarify the mechanism of action, and carry out safety toxicological evaluation to ensure safety and stability, so as to make tannins hopeful to become new drugs on the market.

The structures of 82 tannins from the genus *Terminalia* were reviewed in this paper. The fragmentation pathways of identified tannins were analyzed, and the fragmentation rules of tannins were speculated, which could provide references for the structural analysis of natural medicines and their analogues. In further research, researchers may need to pay more attention to the species and the active substances such as the tannin summarized above.

## Figures and Tables

**Table 1:** Gallotannins **1**–**11** in Figure 1.

No.	Compound name	Source	Reference
**1**	Tri-*O*-galloylshikimic acid	*T. chebula* Retz. (fruits) *T. bellerica* (fruits)	[16]
**2**	1,2,6-Tri-*O*-galloyl-*β*-*D*-glucopyranose	*T. chebula* Retz. (fruits)	[17]
**3**	1,3,6-Tri-*O*-galloyl-*β*-*D*-glucose	*T. citrina* (fruits)	[18]
		*T. chebula* Retz. (fruits)	[19–21]
		*T. catappa* Linn. (the bark)	[22]
		*T. chebula* Retz. (the gall)	[23, 24]
		*T. catappa* Linn. (fruits)	[25]
		*T. chebula* Retz. var. tomentella Kurt. (fruits)	[26]
**4**	3,4,6-Tri-*O*-galloyl-*D*-glucose	*T. chebula* Retz. (fruits)	[19, 27–29]
		*T. horrida* (fruits) *T. chebula* Retz. (fruits)	[16]
**5**	1,2,3-Tri-*O*-galloyl-6-*O*-cinnamoyl-*β*-*D*-glucose	*T. chebula* Retz. (fruits)	[19]
**6**	1,2,3,4-Tetra-*O*-galloyl-*β*-*D*-glucose	*T. chebula* Retz. (fruits)	[30]
**7**	1,3,4,6-Tetra-*O*-galloyl-*β*-*D*-glucose	*T. chebula* Retz. (fruits)	[19, 28]
		*T. bellerica* (fruits) *T. horrida* (fruits) *T. chebula* Retz. (fruits)	[16]
		*T. chebula* Retz. var. tomentella Kurt. (fruits)	[26]
**8**	2,3,4,6-Tetra-*O*-galloyl-*D*-glucose	*T. arjuna* (the bark)	[31]
**9**	1,2,3,6-Tetra-*O*-galloyl-*β*-*D*-glucose	*T. chebula* Retz. (fruits) *T. bellirica* (fruits)	[32]
		*T. chebula* Retz. (fruits)	[19]
		*T. bellirica* (fruits)	[33]
**10**	1,2,3,6-Tetra-*O*-galloyl-4-*O*-cinnamoyl-*β*-*D*-glucose	*T. chebula* Retz. (fruits)	[19]
**11**	1,2,3,4,6-Penta-*O*-galloyl-*β*-*D*-glucose	*T. chebula* Retz. (fruits)	[19, 21, 27, 29, 34]
		*T. chebula* Retz. (fruits) *T. bellirica* (fruits)	[32, 33]
		*T. arjuna* (leaves)	[31]
		*T. horrida* (fruits) *T. chebula* Retz. (fruits) *T. bellerica* (fruits)	[16]
		*T. chebula* Retz.	[35]

**Table 2:** Ellagitannins **12**–**71** in Figure 2.

No.	Compound name	Source	Reference
**12**	Galloyl-free chebulinic acid	*T. chebula* Retz. (fruits)	[36]
**13**	4-*O*-(4″-*O*-Galloyl-*α*-*L*-rhamnopyranosy) ellagic acid	*T. chebula* Retz. (fruits)	[16]
**14**	4′-*O*-Galloy-3,3′-di-*O*-methylellagic acid 4-*O*-*β*-*D*-xylopyranoside	*T. superba* (the bark)	[37]
**15**	Castalin	*T. catappa* Linn. (the bark) *T. parviflora* (the bark)	[22]
**16**	Terflavin D	*T. chebula* Retz. (fruits)	[38]
**17**	2,3-(*S*)-HHDP-6-*O*-galloyl-*D*-glucose	*T. parviflora* (the bark)	[22]
**18**	Corilagin	*T. chebula* Retz. (fruits)	[19, 27, 29, 39–42]
		*T. chebula* Retz. (fruit rinds) *T. bellirica* (fruit rinds)	[32]
		*T. citrina* (fruits)	[18]
		*T. chebula* Retz. (pericarps)	[18]
		*T. chebula* var. parviflora (fruits) *T. chebula* Retz. (fruits)	[43]
		*T. chebula* Retz.	[44]
		*T. catappa* Linn. (leaves)	[45, 46]
		*T. catappa* Linn. (bark)	[22]
		*T. catappa* Linn. (fruits)	[25]
		*T. chebula* Retz. (fruits)	[28, 47]
		*T. bellerica* (fruits) *T. chebula* Retz. (fruits) *T. horrida* (fruits)	[16]
		*T. chebula* Retz. (fruits and bark)	[48]
		*T. ferdinandiana* (fruits)	[49]
		*T. bellerica* (fruits) *T. chebula* Retz. (fruits)	[33]
**19**	Sanguiin H-4	*T. calamansanai* (leaves)	[50, 51]
**20**	Gemin D	*T. chebula* Retz. (fruits)	[19]
**21**	Punicacortein A	*T. catappa* Linn. (fruit peels)	[52]
**22**	Chebulanin	*T. chebula* Retz. (fruits)	[19, 27, 29, 33, 34, 53, 54]
		*T. chebula* Retz. *T. bellirica*	[55]
		*T. catappa* Linn. (leaves)	[45]
		*T. chebula* Retz. var. parviflora (fruits) *T. chebula* Retz. (fruits)	[43]
		*T. brachystemma* (leaves) *T. mollis* (leaves)	[56]
		*T. horrida* (fruits) *T. bellerica* (fruits) *T. chebula* Retz. (fruits)	[16]
**23**	Chebumeinin A	*T. chebula* Retz. (fruits)	[40]
**24**	Chebumeinin B	*T. chebula* Retz. (fruits)	[40, 41]
**25**	4-*O*-(3″,4″-Di-*O*-galloyl-*α*-*L*-rhamnosyl) ellagic acid	*T. catappa* Linn. (leaves)	[45]
		*T. chebula* Retz. (fruits)	[19]
		*T. brownii* (the bark)	[57]
		*T. horrida* (fruits) *T. chebula* Retz. (fruits)	[16]
**26**	4-*O*-(2″,4″-Di-*O*-galloyl-*α*-*L*-rhamnosyl) ellagic acid	*T. chebula* Retz. (fruits)	[19]
**27**	3′-*O*-Methyl-4-*O*-(3″,4″-di-*O*-galloyl-*α*-*L*-rhamnopyranosyl) ellagic acid	*T. chebula* Retz. (fruits)	[16]
**28**	Punicalin	*T. catappa* Linn. (leaves)	[45, 58]
		*T. arjuna* (leaves)	[31]
		*T. chebula* Retz. (fruits)	[38]
		*T. parviflora* (the bark)	[22]
		*T. triflora* (leaves)	[59]
		*T. horrida* (fruits)	[16]
		*T. calamansanai* (leaves)	[51]
**29**	4,6-*O*-Isoterchebuloyl-*D*-glucose	*T. macroptera* (the bark)	[60]
**30**	Pedunculagin	*T. chebula* Retz.	[61]
**31**	Terflavin B	*T. catappa* Linn. (leaves)	[45, 58]
		*T. macroptera* (the bark)	[60]
		*T. chebula* Retz. (fruits)	[38]
		*T. horrida* (fruits)	[16]
**32**	Tercatain	*T. catappa* Linn. (fruit peels)	[52]
		*T. chebula* Retz. (fruits)	[19]
		*T. catappa* Linn. (the bark)	[22]
		*T. catappa* Linn. (leaves)	[45, 46]
**33**	Tellimagrandin I	*T. catappa* Linn. (bark)	[62]
		*T. muelleri* (leaves)	[63]
		*T. chebula* Retz. (fruits)	[19]
		*T. bellerica* (fruits)	[16]
		*T. catappa* Linn. (leaves)	[58]
		*T. calamansanai* (leaves)	[51]
**34**	Sanguiin H-1	*T. calamansanai* (leaves)	[51]
**35**	1,3-Di-*O*-galloyl-2,4-chebuloyl-*β*-*D*-glucose	*T. horrida* (fruits) *T. chebula* (fruits)	[16]
**36**	1,6-Di-*O*-galloyl-2,4-chebuloyl-*β*-*D*-glucose	*T. horrida* (fruits) *T. chebula* Retz. (fruits)	[16]
		*T. chebula* Retz.	[64]
**37**	Castalagin	*T. catappa* Linn. (the bark)	[62]
		*T. parviflora* (the bark) *T. catappa* Linn. (the bark)	[22]
**38**	Terflavin C	*T. chebula* Retz. (fruits)	[38]
**39**	2-*O*-Galloylpunicalin	*T. calamansanai* (leaves)	[50]
		*T. arjuna* (the bark)	[31]
		*T. triflora* (leaves)	[59]
**40**	2,3,4,6-*bis*-Hexahydroxydiphenyl-1-galloyl-*β*-glucose	*T. arjuna* (leaves)	[31]
**41**	Casuarinin	*T. catappa* Linn. (the bark)	[22, 51, 62]
		*T. chebula* Retz. (fruits)	[27, 29, 40, 65]
		*T. arjuna* Linn. (the bark)	[66, 67]
**42**	1(*α*)-*O*-Galloylpedunculagin	*T. calamansanai* (leaves)	[51]
**43**	Tellimagrandin II	*T. catappa* Linn. (the bark)	[62]
		*T. catappa* Linn. (leaves)	[45]
		*T. calamansanai* (leaves)	[51]
**44**	Geraniin	*T. chebula* Retz. (fruits)	[68]
		*T. catappa* Linn. (leaves)	[58]
**45**	Granatin B	*T. catappa* Linn. (leaves)	[58]
**46**	Praecoxin A	*T. calamansanai* (leaves)	[51]
**47**	Terchebin	*T. chebula* Retz var. tomentella Kurt. (fruits)	[26]
**48**	Chebulagic acid	*T. chebula* Retz. (fruits)	[19, 21, 27, 28, 39–41, 43, 53, 68–75]
		*T. chebula* Retz. (fruit rinds) *T. bellirica* (fruit rinds)	[32]
		*T. citrina* (fruits)	[18]
		*T. catappa* Linn. (leaves)	[45, 46, 58]
		*T. chebula* Retz. (pericarps)	[76]
		*T. muelleri* (leaves)	[63]
		*T. chebula* Retz.	[44, 76]
		*T. chebula* Retz. (Galls)	[23, 24]
		*T. bellerica* (fruits) *T. chebula* Retz. (fruits) *T. horrida* (fruits)	[16]
		*T. chebula* Retz. (fruits and bark)	[48]
		*T. arjuna* (leaf, stem, root, bark, fruit) *T. bellerica* (leaf, stem, root, bark, fruit) *T. chebula* Retz. (leaf, stem, root, bark, fruit)	[77]
		*T. bellerica* (fruits)	[33]
**45**	Granatin B	*T. catappa* Linn. (leaves)	[58]
**46**	Praecoxin A	*T. calamansanai* (leaves)	[51]
**47**	Terchebin	*T. chebula* Retz var. tomentella Kurt. (fruits)	[26]
**48**	Chebulagic acid	*T. chebula* Retz. (fruits)	[19, 21, 27, 28, 39–41, 43, 53, 68–75]
		*T. chebula* Retz. (fruit rinds) *T. bellirica* (fruit rinds)	[32]
		*T. citrina* (fruits)	[18]
		*T. catappa* Linn. (leaves)	[45, 46, 58]
		*T. chebula* Retz. (pericarps)	[76]
		*T. muelleri* (leaves)	[63]
		*T. chebula* Retz.	[44, 77]
		*T. chebula* Retz. (Galls)	[23, 24]
		*T. bellerica* (fruits) *T. chebula* Retz. (fruits) *T. horrida* (fruits)	[16]
		*T. chebula* Retz. (fruits and bark)	[48]
		*T. arjuna* (leaf, stem, root, bark, fruit) *T. bellerica* (leaf, stem, root, bark, fruit) *T. chebula* Retz. (leaf, stem, root, bark, fruit)	[78]
		*T.bellerica* (fruits)	[33]
		*T. chebula* Retz var. tomentella Kurt. (fruits)	[26]
**49**	Rugosin B	*T. calamansanai* (leaves)	[51]
**50**	Chebulinic acid	*T. chebula* Retz. (fruits)	[19, 20, 27–30, 36, 39, 43, 53, 65, 68, 70, 73, 79–84]
		*T. chebula* Retz. (fruits) *T. bellirica* Roxb. (fruits)	[32]
		*T. chebula* Linn. (pericarps)	[85]
		*T. chebula* Retz. (pericarps)	[76]
		*T. chebula* Retz.	[44]
		*T. chebula* Retz. (Galls)	[23, 24]
		*T. bellerica* (fruits) *T. chebula* Retz. (fruits) *T. horrida* (fruits)	[16]
		*T. chebula* Retz. (fruits and the bark)	[48]
		*T. arjuna* (leaf, stem, root, bark, fruit) *T. bellerica* (leaf, stem, root, bark, fruit) *T. chebula* Retz. (leaf, stem, root, bark, fruit)	[78]
		*T. bellereica* (fruits) *T. chebula* Retz. (fruits)	[33]
		*T. chebula* Retz var. tomentella Kurt. (fruits)	[26]
**51**	Methyl chebulagate	*T. chebula* Retz. (fruits)	[19]
**52**	Neochebulagic acid	*T. chebula* Retz. (fruits)	[19]
**53**	Neochebulinic acid	*T. chebula* Retz. (fruits)	[27, 29, 43]
		*T. chebula* Retz. var. tomentella Kurt. (fruits)	[26]
**54**	6′-*O*-Methyl neochebulagate	*T. chebula* Retz. (fruits)	[19]
**55**	Methyl neochebulagate	*T. chebula* Retz. (the gall)	[24]
		*T. horrida* (fruits) *T. chebula* Retz. (fruits)	[16]
**56**	Methyl neochebulinate	*T. chebula* Retz. (fruits)	[19]
		*T. horrida* (fruits) *T. chebula* Retz. (fruits)	[16]
		*T. chebula* Retz. var. tomentella Kurt. (fruits)	[26]
**57**	Dimethyl neochebulagate	*T. chebula* Retz. (fruits)	[19]
**58**	Dimethyl 4′-epineochebulagate	*T. chebula* Retz. (fruits)	[19]
**59**	Dimethyl neochebulinate	*T. chebula* Retz. (fruits)	[19]
**60**	Grandinin	*T. catappa* Linn. (the bark)	[22]
**61**	Calamanin A	*T. calamansanai* (leaves)	[51]
**62**	*α*-Punicalagin	*T. oblongata* (leaves)	[86]
		*T. myriocarpa* Heurck (leaves)	[87]
		*T. chebula* Retz. (fruits)	[28]
**63**	*β*-Punicalagin	*T. oblongata* (leaves)	[86]
		*T. myriocarpa* Heurck (leaves)	[87]
**64**	Terchebulin	*T. macroptera* (roots)	[88]
		*T. chebula* Retz. (fruits)	[27, 29, 38]
		*T. laxiflora* (wood)	[89, 90]
**65**	Iso/terchebulin	*T. catappa* Linn. (the bark)	[62]
		*T. macroptera* (the bark)	[60]
		*T. chebula* Retz. (Galls)	[23, 24]
**66**	Terflavin A	*T. catappa* (the bark)	[62]
		*T. macroptera* (the bark)	[60]
		*T. chebula* Retz. (fruits)	[19, 38]
		*T. macroptera* (roots)	[88]
		*T. catappa* Linn. (leaves)	[58]
**67**	Eucalbanin A	*T. muelleri* (leaves)	[63]
**68**	Rugosin A	*T. calamansanai* (leaves)	[51]
**69**	tergallagin	*T. catappa* Linn. (leaves)	[58]
**70**	1-*α*-*O*-Galloylpunicalagin	*T. calamansanai* (leaves)	[50, 51]
**71**	Calamansanin	*T. calamansanai* (leaves)	[51]

**Table 3:** Hydrolysable tannin polymers **72**–**74** in Figure 3.

No.	Compound name	Source	Reference
**72**	Castamollinin	*T. catappa* Linn. (the bark)	[22]
**73**	Calamanin B	*T. calamansanai* (leaves)	[51]
**74**	Calamanin C	*T. calamansanai* (leaves)	[51]

**Table 4:** Condensed tannins **75**–**79** in Figure 4.

No.	Compound name	Source	Reference
**75**	Procyanidin B1	*T. tomentosa* (the bark)	[91]
		*T. catappa* Linn. (the bark)	[22]
**76**	Procyanidin B2	*T. tomentosa* (the bark)	[91]
**77**	Procyanidin B3	*T. tomentosa* (the bark)	[91]
**78**	3′-*O*-Galloyl procyanidin B-2	*T. catappa* Linn. (the bark)	[22]
**79**	Procyanidin C1	*T. tomentosa* (bark)	[91]

**Table 5:** Complex tannins **80**–**82** in Figure 5.

No.	Compound name	Source	Reference
**80**	Catappanin A	*T. catappa* Linn. (the bark)	[22]
**81**	Acutissimin A	*T. catappa* Linn. (the bark)	[22]
**82**	Eugenigrandin A	*T. catappa* Linn. (the bark)	[22]

**Table 6:** The MS spectral data of compounds **1**–**82** except those which have no reported MS data.

No.	Compound name	Molecular formula	Ion source	[M-H]^–^	Fragments	Reference
**1**	Tri-*O*-galloylshikimic acid	C_28_H_22_O_17_	ESI	628.9	477 (15), 325 (1), 169 (100)	[16]
**2**	1,2,6-Tri-*O*-galloyl-*β*-*D*-glucose	C_27_H_24_O_18_		635	465 (100), 313 (20), 169 (10)	[92]
				635.093	465.0479, 313.0427, 169.0061	[93]
**3**	1,3,6-Tri-*O*-galloylglucose	C_27_H_24_O_18_		635.0895	465.06714 [C_20_H_17_O_13_]^−^, 211.02463 [C_9_H_7_O_6_]^−^, 169.01404 [C_7_H_5_O_5_]^−^, 125.02427 [C_6_H_5_O_3_]^−^	[94]
**5**	3,4,6-Tri-*O*-galloyl-*D*-glucose	C_27_H_24_O_18_	ESI	635.0882	169 (9), 235 (2), 271 (4), 295 (14), 313 (9), 405 (5), 423 (30), 465 (68), 483 (100), 617 (11)	[95]
**6**	1,2,3,4-Tetra-*O*-galloyl-*β*-*D*-glucose	C_34_H_28_O_22_	ESI	787	617, 393, 169	[32]
**7**	1,3,4,6-Tetra-*O*-galloyl-*β*-*D*-glucose	C_34_H_28_O_22_		787	635, 617	[96]
**8**	2,3,4,6-Tetra-*O*-galloyl-*D*-glucose	C_34_H_28_O_22_	ESI	787	617, 635	[97]
			ESI	787.0914	635.0902, 617.0795,465.0709	[98]
				787.0989	169.0158, 295.0297, 313.0570, 447.1352, 465.1383, 483.0638, 617.1949, 635.2112	[99]
				787.0996	617.0902 [M-H-GA]^−^, 447.0732 [M-H-2GA]^−^, 295.0418 [M-H-2GA-C_7_H_4_O_4_]^−^, 169.0140 [GA-H]^−^	[100]
			ESI	787.1079	617.0834, 465.0731, 313.0606, 169.0177	[101]
			ESI	787	635 [M-H-152]^−^, 617 [M-H-170]^−^, 483 [M-H-304]^−^, 465 [M-H-322]^−^, 447 [M-H-340]^−^, 169 [GA-H]^−^	[102]
**9**	1,2,3,6-Tetra-*O*-galloyl-*β*-*D*-glucose	C_34_H_28_O_22_	ESI	787.0986	295 (1), 403 (2), 421 (0.4), 429 (1), 447 (2), 465 (3), 529 (0.2), 573 (4), 617 (100), 635 (31)	[95]
**11**	1,2,3,4,6-Penta-*O*-galloyl-*β*-*D*-glucose	C_41_H_32_O_26_	ESI	939.1101	329 (0.4), 439 (0.4), 447 (0.2), 515 (0.2), 599 (1), 601 (0.2), 617 (3), 725 (1), 769 (100), 787 (8)	[95]
				939.111	787.1282 [M-H-C_7_H_4_O_4_]^−^, 769.1003 [M-H-GA]^−^, 617.0884 [M-H-GA-C_7_H_4_O_4_]^−^, 447.0593 [M-H-2GA-C_7_H_4_O_4_]^−^, 259.0248 [M-H-4GA]^−^, 169.0140 [GA-H]^−^	[103]
			ESI	939	769[M-H-GA]^–^, 617[M-H + H2O-2GA]^–^	[104]
			ESI	939.11090	769.1, 617.1, 465.1, 447.1, 295.0, 169.0	[105]
			ESI	939	769, 787, 617	[97]
			ESI	939	939[M-H]^−^, 769[M-H-GA]^−^, 617[M-H + H_2_O-2GA]^−^, 447[M-H + H_2_O-3GA]^−^, 169[GA]^−^, 125[GA-CO_2_]^−^	[50]
			ESI	939	787, 769, 617, 599, 447	[106]
				939.112	169, 617, 769	[107]
				939	469, 769, 629, 617, 465, 313, 169, 125	[108]
			ESI	939	787, 769, 635, 617	[109]
			ESI	939.1104	787 [M-H-C_7_H_4_O_4_]^−^, 769 [M-H-C_7_H_6_O_5_]^−^, 635 [M-H-C_14_H_8_O_8_]^−^, 617 [M-H-C_14_H_10_O_9_]^−^, 465 [M-H-C_21_H_14_O_13_]^−^, 447 [M-H-C_21_H_16_O_14_]^−^, 313 [M-H-C_28_H_18_O_17_]^−^, 295 [M-H-C_28_H_19_O_18_]^−^, 169 [M-H-C_34_H_26_O_21_]^−^, 125 [C_35_H_26_O_23_]^−^	[110]
			ESI	939.3	169.0, 393.1, 769.2	[111]
**13**	4-*O*-(4″-*O*-Galloyl-*α*-*L*-rhamnopyranosyl) ellagic acid	C_27_H_20_O_16_	ESI	599	447 (23), 429 (2), 301 (100), 297 (6), 169 (3)	[16]
**15**	Castalin	C_27_H_20_O_18_		631	613 (100)	[112]
			ESI	631.1	301 [EA-H]^–^, 331.0 [Galloylglu-H]^−^, 481.0 [HHDP-glu-H]^−^	[113]
				631	479, 317, 301	[114]
			ESI	631.0586	461.033 (71) [M-H–C_7_H_4_O_4_–H_2_O]^−^, 445.0461 (17) [M-H-C_7_H_4_O_5_-H_2_O]^−^, 300. 9986 (78) [ellagic acid]^−^, 273.0030, 245.0092 (44), 229.0142(45), 169.0143 (100) [GA]^−^, 125.0254 (30)	[115]
**18**	Corilagin	C_27_H_22_O_18_		633.0734	470.9841	[116]
				633.0762	463.0793, 300.9986, 169.0133	[117]
				633.0725	463 (7), 301 (100), 275 (30), 245 (5), 169 (7), 125 (4)	[118]
			ESI	633	476, 454	[32]
**19**	Sanguiin H-4	C_27_H_22_O_18_	ESI	633.0719	327, 343, 177	[119]
			ESI	633	481, 301, 275, 249, 635, 617, 465, 447, 353, 339, 321, 315, 303, 277, 257, 229, 211, 259, 231	[120]
**22**	Chebulanin	C_27_H_24_O_19_		651	633, 481, 463, 291, 275	[100]
				651	481 [M-galloyl]^−^, 651 [M-H]^−^, 1303 [2M-H]^−^	[121]
				651	633 [M-H-H_2_O]^−^, 405, 300, 275	[122]
**23**	Chebumeinin A	C_29_H_30_O_18_		669	366.9	[123]
**24**	Chebumeinin B	C_28_H_28_O_19_		669	366.8	[123]
**25**	4-*O*-(3″,4″-Di-*O*-galloyl-*α*-*L*-rhamnopyranosyl) ellagic acid	C_34_H_24_O_20_	ESI	751.1	599 (22), 581 (6), 449 (30), 411 (4), 300 (100), 297 (8), 169 (6),151 (2)	[16]
**27**	3′-*O*-Methyl-4-*O*-(3″,4″-di-*O*-galloyl-*α*-*L*-rhamnopyranosyl) ellagic acid	C_35_H_26_O_20_	ESI	765.2	613 (32), 595 (100), 461 (5), 449 (30), 443 (41), 425 (10), 315 (31), 169 (56)	[16]
**28**	Punicalin	C_34_H_22_O_22_		781	601, 301	[124]
			ESI	781.0531	721, 601, 271	[125]
			ESI	781.5	299.4	[126]
				781	721, 601, 557, 451, 299	[100]
				781	601, 299	[127]
**30**	Pedunculagin	C_34_H_24_O_22_		783.0673	300.9975	[116]
			ESI	783.07	1567.14 [2M-H]^−^, 391.03 [M-2H]^2–^	[128]
			ESI	783	481, 301, 257	[129]
			ESI	783	391 [M-2H]^2–^, 783 [M-H]^−^, 1567 [2M-H]^–^	[121]
			ESI	783.2	783.2, 481.1, 301.0	[130]
			ESI	783.0686	481.0516, 300, 9975	[131]
				783	481, 301, 244	[114]
				783.068	481, 301, 275	[125]
			ESI	783.0692	935.0790, 613.0463, 300.9990	[132]
			ESI	783.0679	481, 301	[133]
				783.0699	481.0606, 391.0307,300.9999, 275.0191	[134]
				783	301, 481, 275	[97]
			ESI	783.06	481.06, 301.00, 275.02	[135]
**31**	Terflavin B	C_34_H_24_O_22_	ESI	783	631 (11), 451 (100), 299 (1)	[16]
**33**	Tellimagrandin I	C_34_H_26_O_22_	ESI	785.08	301.00, 275.02, 169.01	[135]
					784.6, 450.9, 402.6, 391.7, 214.7	[136]
			ESI	785	301, 483, 615	[137]
			ESI	785	301, 483, 633, 615, 463, 419	[97]
			ESI	785.0836	301, 483, 633	[133]
			ESI	785.0866	633, 481,301, 275, 222	[138]
			ESI	785	392 [M–2H]^2–^, 785 [M–H]^−^, 1571 [2M–H] ^−^	[121]
			ESI	785.084	633.07, 615.06, 483.08, 300.99, 275.02	[139]
			ESI	785	615,483,301	[129]
**35**	1,3-Di-*O*-galloyl-2,4-chebuloyl-*β*-*D*-glucose	C_34_H_28_O_23_	ESI	802.9	337 (100), 319 (47), 293 (41), 275 (61), 169 (8)	[16]
**37**	Castalagin	C_41_H_26_O_26_	ESI	933	915, 631, 451, 301	[140]
			ESI	933.0644	915.0509, 631.0575, 479.0464, 461.0377, 300.9991	[141]
			ESI	933	915, 631, 613, 569, 493, 301	[142]
					915, 783, 631, 613, 569, 467, 493, 323, 301, 146	
			ESI	933	915 (95), 631 (100), 425 (20), 301 (5)	[112]
				933	181.1, 466.0	[113]
			ESI	933.0649	466.0299, 300.9968	[134]
			ESI	933	915, 631, 613, 569	[106]
				933	915, 871, 569, 301	[114]
			ESI	933.1	783.1, 631.1, 451.1, 301.0	[130]
			ESI		466 [M-2H]^2–^, 933 [M-H]^–^, 933 [2M-2H]^2–^, 1867 [2M-H]^–^	[121]
			ESI		935, 915, 613, 301	[143]
			ESI	933	631, 451, 301	[144]
**39**	2-*O*-Galloylpunicalin	C_41_H_26_O_26_		933	781, 721, 601	[124]
**41**	Casuarinin	C_41_H_28_O_26_	ESI	935.0796	785.1, 633.1, 483.1, 451.0, 425.0, 301.0, 275.0, 169.0	[105]
			ESI	935	917, 633, 783, 301	[137]
			ESI	935	467 [M-2H]^2–^, 935 [M-H]^–^, 1871 [2M-H]^–^	[121]
				935.076	633.075, 300.9999	[145]
**43**	Tellimagrandin II	C_41_H_30_O_26_	ESI	937.0953	301.0, 275.0, 249.0, 169.0	[105]
			ESI	937	767, 741, 465, 301	[97]
			ESI	937	785, 767, 635, 465, 301	[106]
			ESI	937.0945	785, 633, 483, 301, 278, 237	[138]
**44**	Geraniin	C_41_H_28_O_27_	ESI	951.0747	907.0849, 781.0537, 605.0788, 479, 425.0251, 298, 273.0042	[141]
			ESI	951.0762	933.0717 (100) [M-H-H_2_O]^–^, 300.9991 (52), 169.0141 (2)	[115]
				951.6751	463.0505, 301.9987, 273.0040, 169.0132	[146]
			ESI	951	457 [M-2H_2_O-2H]^2–^, 466 [M-H_2_O-2H]^2–^, 951 [M-H]^–^, 1903 [2M-H]^–^	[121]
			ESI	951.0721	933.0770 [M-H-H_2_O]^–^, 300.9990, 169.0144	[147]
			ESI	951.07	951.07 [M-H]^–^, 466.03 [M-2H]^2–^, 300.99 [EA-H]^–^, 633.07 [M-318-H]^–^	[128]
**45**	Granatin B	C_41_H_27_O_27_	ESI	951.0719	933 (7), 463 (20), 301 (100), 273 (32), 245 (17), 229 (3), 167 (3)	[118]
			ESI	951	933, 915, 301	[148]
			ESI	951.0745	933.0604, 613.2044, 300.9980	[131]
**46**	Praecoxin A	C_41_H_28_O_27_	ESI	951	783, 605, 889, 481, 301	[149]
**48**	Chebulagic acid	C_41_H_30_O_27_	ESI	953	476, 169	[32]
				953	935, 807, 633, 481, 463, 319, 301	[100]
			ESI	953	476 [M-2H]^2–^, 953 [M-H]^–^	[121]
**49**	Rugosin B	C_41_H_30_O_27_	ESI	953.0902	909.1, 785.1, 766.1, 597.0, 301.0, 275.0, 249.0, 169.0	[105]
				953.2	909 (100), 883 (1), 785 (5)	[150]
**50**	Chebulinic acid	C_41_H_32_O_27_		955	477 [M-2H]^2–^, 169	[32]
				955	937, 803, 785, 641, 607, 465, 337, 275, 131	[100]
				955	477 [M-2H]^2–^, 955 [M-H]^–^	[121]
				955.1018	785, 169	[151]
**52**	Neochebulagic acid	C_41_H_32_O_28_		971	953 [M-H-H2O]^–^, 935 [M-H-H_2_O-H_2_O]^–^, 467 [M-2H-H_2_O-H_2_O]^2–^, 301	[122]
**56**	Methyl neochebulinate	C_42_H_36_O_28_	ESI	987.2	635 (100), 465 (1), 351 (3), 169 (1)	[16]
**60**	Grandinin	C_46_H_34_O_30_	ESI	1065	1047 (50), 1029 (50), 975 (100),	[112]
**62**	*α*-Punicalagin	C_48_H_27_O_30_	ESI	1083.056	781 (40), 601 (35), 575 (20), 301 (100), 275 (7), 249 (5)	[118]
				1083	781 (60), 601 (100), 575 (22)	[152]
				1083.059	781.6071, 601.3680, 301.4796	[131]
			ESI	1083	781, 541, 301	[153]
**63**	*β*-Punicalagin	C_48_H_27_O_30_	ESI	1083.054	1083 (43), 781 (55), 719 (29), 601 (86), 575 (29), 301 (100), 275 (43), 249 (15)	[118]
				1083	781 (35), 601 (100), 575 (15)	[152]
				1083.059	781.6071, 601.3680, 301.4796	[131]
			ESI	1083	781, 541, 301	[153]
**67**	Eucalbanin A	C_48_H_30_O_30_	ESI	1085	765, 633, 473	[137]
			ESI	1085	933, 783, 765, 739, 633, 597, 469, 407	[97]
			ESI	1085.074	783.07, 633.07, 450.99, 300.99	[139]
**68**	Rugosin A	C_48_H_34_O_31_	ESI	1105.101	530.0, 891.1, 301.0, 169.0	[105]
			ESI	1105.3	1061 (100), 937 (5), 935 (10), 917 (3)	[150]
**75**	Procyanidin B1	C_30_H_26_O_12_		577.1344	577, 451, 425, 407, 289, 245, 161, 125	[154]
				577.16	287, 289, 425, 451	[155]
			ESI	577.1351	425.0875 (100), 451.1030 (90), 289.0713 (60), 407.0767 (60), 299.0556 (30), 287.0557 (10)	[156]
**76**	Procyanidin B2	C_30_H_26_O_12_		577.152	287, 289, 425, 451	[155]
			ESI	577	451 (23.7), 425 (100), 407 (69.6), 289 (29.0), 408 (17.7), 407 (100), 289 (100), 281 (85.7), 256	[157]
**77**	Procyanidin B3	C_30_H_26_O_12_	ESI	577.1331	407 (75), 289 (81), 245 (67)	[158]
			ESI	577.1375	425, 407, 289, 287	[159]
**78**	3′-*O*-Galloyl procyanidin B2	C_37_H_30_O_16_		729.1458	407.0766, 289.0716	[160]
				729.1471	303.05055, 364.58214, 441.08203	[161]
**79**	Procyanidin C1	C_45_H_38_O_18_	ESI	865.1964	739.1640, 575.1171	[162]
			ESI	865.195	865 (37), 695 (100), 577 (1), 407 (64), 289 (42)	[158]
			MALDI	865.191	287, 289, 575, 577, 713, 425, 739, 451, 413	[155]
			ESI	865	675.3, 528.6	[163]
			ESI	865.1984	739, 713, 577, 289	[119]
			ESI	865.1985	739.1722, 577.1378, 451.1054, 407.0793, 287.0575, 245.0460	[164]
**81**	Acutissimin A	C_56_H_38_O_31_	ESI	1205	1205, 915, 613, 602, 301	[143]
